# Thromboembolic risks associated with paroxysmal and persistent atrial fibrillation in Asian patients: a report from the Chinese atrial fibrillation registry

**DOI:** 10.1186/s12872-019-1260-7

**Published:** 2019-11-27

**Authors:** Ying Wang, Chang-Sheng Ma, Xin Du, Liu He, Jie Li, Guo-Hong Wang, Dan Wen, Jian-Zeng Dong, Jian-Hong Pan, Gregory Y. H. Lip

**Affiliations:** 1 Department of Cardiology, Beijing Anzhen Hospital, Capital Medical University, National Clinical Research Center for Cardiovascular Diseases, Beijing, China; 2 Cardiovascular Center, Beijing Tongren Hospital, Capital Medical University, Beijing, China; 3 Biostatistics Department, Peking University Clinical Research Institute, Beijing, China; 4 Liverpool Centre for Cardiovascular Science, University of Liverpool and Liverpool Heart & Chest Hospital, Liverpool, UK; 5 Aalborg Thrombosis Research Unit, Department of Clinical Medicine, Aalborg University, Aalborg, Denmark

**Keywords:** Atrial fibrillation, Thromboembolism, Risk factors, Stroke, Outcome

## Abstract

**Background:**

Several studies have reported on atrial fibrillation (AF) outcomes, including thromboembolism in patients with paroxysmal and non-paroxysmal AF; however the findings still remain controversial on whether risks differ between these clinical subtypes and limited data are available in Asian cohorts.

**Methods:**

We compared the risk of thromboembolism between paroxysmal and persistent AF patients, in a large contemporary Chinese cohort study. A total of 8529 non-valvular atrial fibrillation (NVAF) patients from the Chinese Atrial Fibrillation Registry (CAFR) study were enrolled. The study subjects were divided into two groups: paroxysmal AF (PaAF, defined as AF lasting within 7 days, *n* = 4642) and persistent AF (PeAF, lasting over 7 days, *n* = 3887) groups.

**Results:**

In non-anticoagulated patients, PeAF group demonstrated a higher risk of stroke, all-cause death, cardiac/ non-cardiac death and composition of stroke/ transient ischemic attack (TIA)/peripheral thromboembolism (PT)/all-cause death, compared to the PaAF group. No significant difference was found in anticoagulated subjects. On multivariate analysis in non-anticoagulated patients, age ≥ 75 years (*P* = 0.046) and prior stroke/TIA/PT (*P* = 0.018) but not AF type (*P* = 0.63) were significantly associated with the risk of stroke/TIA/PT events.

**Conclusions:**

Stroke, all-cause death and cardiac/non-cardiac death in Chinese NVAF population was increased in non-anticoagulated PeAF patients compared with PaAF group, but same between anticoagulated PeAF and PaAF patients. After adjustment, AF type was not an independent predictor of thromboembolism in NVAF patients.

**Clinical trial registration:**

Chinese Clinical Trial Registry ChiCTR-OCH-13003729. Registered 22 October 2013.

## Background

Atrial fibrillation (AF) is the most common sustained cardiac arrhythmia worldwide and is strongly associated with the risk of stroke, thromboembolism and death [1]. The risks of thromboembolism are dependent on various clinical risk factors for stroke [2]. Current guidelines recommend oral anticoagulation (OAC) in high risk patients, irrespective of whether the AF pattern is paroxysmal or persistent [3, 4]. Nevertheless, higher AF burden and a more sustained AF pattern have been associated with a greater risk of thromboembolism [5–13], although other studies reported opposite findings [14–19]. The inconsistency may be possibly due to different sample sizes enrolled among previous studies, as well as smaller event numbers due to OAC use. Also, limited data are available from Asian population.

The Chinese Atrial Fibrillation Registry (CAFR) is a large contemporary Chinese cohort study, documenting the clinical epidemiology and outcomes in Chinese patients with AF. In this report from CAFR, we compared the risk of thromboembolism between paroxysmal and persistent AF patients.

## Methods

The rationale and design of the CAFR study have been reported previously [20, 21]. In brief, CAFR is a prospective, multicenter, hospital-based ongoing registry conducted in Beijing, China. Ethics approval was obtained from the institutional review committee of Beijing Anzhen Hospital. Demographic data, comorbidities, treatments received and results of laboratory examinations of each patient were recorded after the patient signed the consent form. Every patient was followed up by outpatient procedures or by telephone at the time of 3 months, 6 months and every 6 months thereafter. Major events including ischemic stroke, systemic embolism and bleeding were recorded during follow-up. After excluding those who received successful radiofrequency catheter ablation therapy at baseline and during follow-up, data of 8529 NVAF patients collected between August 2011 and June 2015 were used for the present analyses.

Paroxysmal AF was defined as with spontaneous termination or with intervention within 7 days of onset, although episodes may recur with variable frequency. Persistent AF was defined as AF lasting>7 days. Because of the sustained AF status of longstanding persistent AF and permanent AF defined in AF guidelines, the two patterns of AF were assigned to the persistent group in our study, using a simplified scheme from Levy et al. [22]. AF type of each patient was kept consistent with that of baseline regardless of later changes during follow-up.

The clinical outcomes included the occurrence of fatal and non-fatal ischemic stroke, transient ischemic attack (TIA), other non-central nervous system (CNS) peripheral thromboembolism (PT), intracranial hemorrhage, all-cause death, cardiac death, non-cardiac death and composite outcomes of stroke/TIA/ PT. Endpoint events were adjudicated by neurologists according to the patients’ medical records.

Baseline data of patients in different AF subtypes were reported as mean ± standard deviation or median (25th, 75th percentiles) for continuous variables and frequencies and percentages for categorical variables. Between-group comparisons were performed using Wilcoxon rank-sum tests for continuous variables and Pearson’s chi-squared tests for categorical variables.

Kaplan–Meier curves were plotted for time to clinical events in the two AF groups with or without warfarin or other oral anticoagulants in order to avoid the confounding effect of anticoagulation treatment on thromboembolism events. Cumulative incidence rates were compared with the log-rank test by groups. Multivariate Cox proportional hazards regression model was used to analyze the independent risk factors for stroke/TIA/PT [23, 24] and to assess the association between AF type and stroke risk, adjusted for AF types and components of CHA_2_DS_2_-VASc score: congestive heart failure, hypertension, age ≥ 75 years, age 65-74 years, diabetes mellitus, previous history of stroke/TIA/PT, vascular diseases and female gender. The proportional hazard assumption was assessed using supremum test. *P* value < 0.05 was regarded as statistically significant. All tests of significance were two-sided. All statistical analysis was made using SAS version 9.4 (SAS Institute, Cary, NC, USA).

## Results

There were 4642 patients with PaAF (54.43%) and 3887 patients with PeAF (46.88%) included for the current analysis (Table 1). Compared with PeAF patients, PaAF patients were younger, had smaller left atria (LA) diameter, and had higher creatinine clearance rate (*P*<0.001). PeAF group had a longer history of AF and higher CHADS_2_ and CHA_2_DS_2_-VASc scores. At baseline, PeAF patients had greater prevalence of prior history with hypertension, stroke/TIA/PT, heart failure, diabetes, myocardial infarction and peripheral artery diseases (*P*<0.001), with no difference in history of other coronary diseases and thyroid diseases. More patients with left ventricular ejection fraction (LVEF) less than 40% were in PeAF group compared with PaAF group (*P*<0.001). Significant higher proportions of patients with age ≥ 75 years, female sex, CHADS _2_ and CHA_2_DS_2_-VASc scores ≥2 were observed.

**Table 1:** Characteristics of patients with paroxysmal and persistent AF at study entry

Characteristics	PaAF (*n* = 4642)	PeAF (*n* = 3887)	*P* value
Age (y, mean ± SD)^a^	66.8 ± 12.12	69.1 ± 11.23	0.000
Age group			
65–74 years, n (%)^a^	1294 (49.83%)	1078 (44.20%)	< 0.001
≥ 75 years, n (%)	1303 (50.17%)	1361 (55.80%)	
Gender, female, n(%)	2007 (43.24%)	1542 (39.67%)	< 0.001
BMI (kg/m2, mean ± SD)	25.1 ± 3.69	25.4 ± 3.69	< 0.001
SBP (mmHg, mean ± SD)	129.2 ± 17.17	128.6 ± 17.76	0.114
DBP (mmHg, mean ± SD)	76.9 ± 10.67	78.4 ± 11.38	0.000
LA diameter (mm, mean ± SD)	39.1 ± 6.72	44.9 ± 7.09	0.000
Duration of AF history (y, mean ± SD)	4.6 ± 6.19	6.8 ± 7.40	0.000
Ccr (ml/min, mean ± SD)	78.0 ± 33.13	74.3 ± 33.65	< 0.001
CHADS _2_ score (mean ± SD)	1.7 ± 1.37	2.1 ± 1.46	0.000
CHADS _2_ score n(%)			
0	909 (19.58%)	503 (12.94%)	< 0.001
1	1514 (32.62%)	1017 (26.16%)	
≥ 2	2219 (47.80%)	2367 (60.90%)	
CHA_2_DS_2_-VAS_C_ score (mean ± SD)	2.9 ± 1.89	3.3 ± 1.96	0.000
CHA_2_DS_2_-VAS_C_ score n(%)			
0	447 (9.63%)	255 (6.56%)	< 0.001
1	764 (16.46%)	515 (13.25%)	
≥ 2	3431 (73.91%)	3117 (80.19%)	
LVEF(%, mean ± SD)	63.0 ± 9.31	58.9 ± 11.29	0.000
Comorbidities n(%)			
Hypertension	3156 (68.11%)	2729 (70.35%)	0.025
Congestive heart failure	296 (6.38%)	783 (20.16%)	< 0.001
LVEF			
≥ 40%	2993 (97.27%)	2552 (92.67%)	< 0.001
0–40%	84 (2.73%)	202 (7.33%)	
Diabetes	1137 (24.49%)	1071 (27.55%)	0.001
Prior stroke/TIA/PT	819 (17.64%)	885 (22.78%)	< 0.001
Prior myocardial infarction	262 (5.65%)	259 (6.67%)	0.049
Other coronary artery diseases	815 (17.57%)	647 (16.66%)	0.269
Peripheral artery diseases	34 (1.75%)	44 (3.18%)	0.007
Thyroid diseases	153 (7.75%)	100 (7.06%)	0.452
Baseline medication n(%)			
Aspirin	2220 (47.96%)	1662 (42.86%)	< 0.001
Warfarin/NOAC	1363 (29.36%)	1808 (46.51%)	< 0.001
β-blockers	2493 (53.71%)	2291 (58.94%)	< 0.001
Digoxin	387 (8.34%)	1374 (16.11%)	< 0.001
Amiodarone	502 (10.81%)	130 (3.34%)	< 0.001
Statins	1754 (37.89%)	1551 (39.98%)	0.049
ACEI/ARBs	1705 (36.73%)	1755 (45.15%)	< 0.001

*AF* indicates atrial fibrillation, *PaAF* paroxysmal atrial fibrillation, *PeAF* persistent atrial fibrillation, *SD* standard deviation, *TIA* transient ischemic attack, *PT* peripheral thromboembolism, *BMI* body mass index, *SBP* systolic blood pressure, *DBP* diastolic blood pressure, *LA* left atria, *Ccr* creatinine clearance rate, *LVEF* left ventricular ejection fraction, *NOAC* new oral anticoagulantion, *PT* peripheral thromboembolism, *ACEI/ARB* angiotensin converting enzyme inhibitors/angiotensin receptor blockers, *CHADS*_*2*_ congestive heart failure, hypertension, age 75 years or more, diabetes mellitus and stroke, and *CHA*_*2*_*DS*_*2*_*-VASc* congestive heart failure, hypertension, age 75 years or more, diabetes mellitus, stroke, vascular disease, age 65–74 years and sex category

^a^Data given as n (%) or mean ± SD

Medical therapy at baseline in the two patient groups are listed in Table 1. Approximately 30% of patients with paroxysmal AF and 46.5% of patients with persistent AF were on warfarin or new oral anticoagulants (NOACs). Patients of PeAF group were more likely to be taking rate control medicines, including beta-blockers and digoxin, while PaAF group were more frequently treated with amiodarone. More patients in PeAF group were taking angiotensin converting enzyme inhibitors/angiotensin receptor blockers (ACEI/ARBs) drugs in comparison to those in PaAF group, possibly as a consequence of higher prevalence of congestive heart failure in PeAF group.

Incidence rates of thromboembolic events according to AF types were shown in Table 2, stratified by application of oral anticoagulation drugs. For AF patients not on anticoagulant therapy, the incidences of stroke/TIA/PT were 1.9 vs. 1.3 per 100 patient years for PeAF and PaAF, respectively (*P*<0.003). Likewise, risk of all-cause death and cardiac/non-cardiac death was higher in PeAF patients.

**Table 2:** Thromboembolic outcomes in different groups of atrial fibrillation type stratified by anticoagulant drugs during follow-up

Thromboembolic outcomes	With warfarin/NOAC		HR(95%CI)	*P* value ^a^	Without warfarin/NOAC		HR (95%CI)	*P* value^a^
	Total events/n	Incidence rates/100 pt.^a^yrs			Total events/n	Incidence rates/100 pt.^a^yrs		
Stroke /TIA/PT								
PeAF	51/1804	1.2	0.988	0.955	100/2078	1.9	1.521	0.003
PaAF	37/1359	1.3	(0.647–1.509)		99/3276	1.3	(1.152–2.008)	
Stroke								
PeAF	41/1805	1.0	1.014	0.953	72/2078 77/3276	1.4	1.414	0.003
PaAF	29/1360	1.0	(0.633–1.630)			1.0	(1.025–1.950)	
TIA								
PeAF	11/1807	0.3	0.988	0.980	33/2079	0.6	2.301	0.003
PaAF	8/1362	0.3	(0.397–2.457)		21/3279	0.3	(1.331–3.977)	
PT								
PeAF	2/1808	0.0	0.470	0.408	8/3279	0.1	0.919	0.882
PaAF	3/1363	0.1	(0.078–2.814)		5/2079	0.1	(0.300–2.810)	
Intracranial hemorrhage								
PeAF	9/1808	0.2	1.806	0.875	5/2079	0.1	1.059	0.923
PaAF	6/1363	0.2	(0.386–3.054)		7/3278	0.1	(0.336–3.337)	
All-cause death								
PeAF	57/1806	1.4	1.242	0.327	201/2068	4.0	3.028	< 0.0001
PaAF	33/1361	1.2	(0.805–1.916)		104/3271	1.3	(2.389–3.836)	
Cardiac death								
PeAF	21/1807	0.5	1.603	0.237	101/2073	1.9	4.314	< 0.0001
PaAF	10/1362	0.3	(0.734–3.503)		36/3278	0.5	(2.949–6.312)	
Non-cardiac death								
PeAF	30/1808	0.7	1.17	0.599	68/2078 47/3275	1.3	2.18	< 0.0001
PaAF	18/1362	0.6	(0.652–2.099)			0.6	(1.503–3.162)	
Stroke /TIA/PT/all-cause death								
PeAF	103/1802	2.6	1.119	0.478	285/2067191/3268	5.9	2.352	< 0.0001
PaAF	66/1357	2.4	(0.820–1.527)			2.5	(1.958–2.825)	

*NOAC* indicates new oral anticoagulation, *HR* hazard ratio, *CI* confidence interval, *TIA* transient ischemic attack, *PT* peripheral thromboembolism, *PaAF* paroxysmal atrial fibrillation, and *PeAF* persistent atrial fibrillation

^a^Incidence rates were compared by Cox proportional hazards regression models, stratified by anticoagulant drugs

**Fig. 1:**
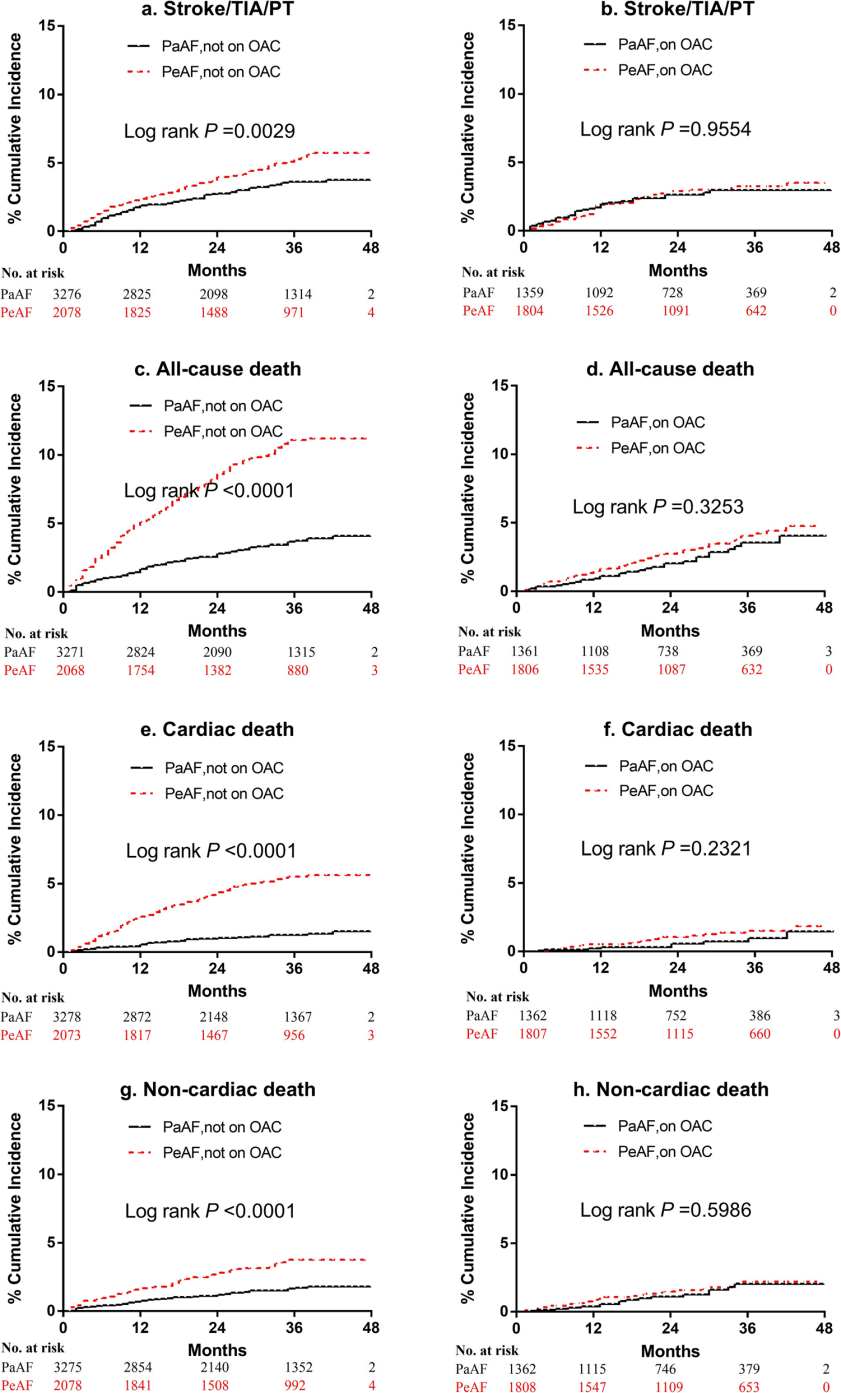
Kaplan-Meier curves for occurrence of outcomes in PeAF vs. PaAF patients with or without OAC. **a**, **b** Stroke/TIA/PT; **c**, **d** all-cause death; **e**, **f** cardiac death; **g, h** non-cardiac death

Kaplan-Meier curves for PeAF vs. PaAF patients with or without OAC for outcomes of stoke/TIA/PT, all-cause death, cardiac death, non-cardiac death, are shown in Fig. 1. For patients not on OAC, PaAF group exhibited significantly lower HRs than PeAF group in risk of stroke/TIA/PT (*P* < 0.003), all cause death (*P* < 0.0001), cardiac death (*P* < 0.0001) and non-cardiac death (*P* < 0.0001). For patients on OAC, clinical outcomes aforementioned were similar in PaAF as in PeAF patients (*P* = 0.955, *P* = 0.327, *P* = 0.237, *P* = 0.599, respectively).

In patients not on anticoagulation, univariate analysis demonstrates that AF type and components of CHA_2_DS_2_-VASc score except for the history of diabetes and female sex were associated with stroke/TIA/PT events (Table 3). On multivariate Cox proportional hazards regression models, age ≥ 75 years [HR 2.10 (1.01–4.360), *P* = 0.046)] and prior stroke/TIA/PT [HR 1.86 (1.11–3.11), *P* = 0.018)] but not AF type [HR 1.13 (0.69–1.86), *P* = 0.63] were independently associated with the risk of stroke/TIA/PT events.

**Table 3:** Univariate and multivariate analysis: stroke/TIA/PT risk factors in NVAF patients not on anticoagulants

Risk factors	Univariate		Multivariate	
	HR (95%CI)	*P* value	HR (95%CI)	*P* value
PeAF vs PaAF	1.52 (1.15–2.00)	0.0031	1.13 (0.69–1.86)	0.63
Congestive heart failure	1.54 (1.09–2.17)	0.014	1.00 (0.55–1.82)	0.99
Age (≥75ys)	3.56 (2.38–5.35)	< 0.0001	2.10 (1.01–4.36)	0.046
Age (65-74ys)	2.06 (1.30–3.25)	0.002	1.38 (0.62–3.08)	0.44
Hypertension	1.84 (1.30–2.61)	0.0005	1.39 (0.71–2.74)	0.34
Diabetes	1.00 (0.73–1.39)	0.996	0.82 (0.48–1.39)	0.46
Prior stroke/TIA/PT	2.51 (1.87–3.37)	< 0.0001	1.86 (1.11–3.11)	0.018
Female	1.15 (0.87–1.53)	0.31	1.44 (0.89–2.34)	0.14
Vascular diseases	2.43 (1.42–4.16)	0.001	1.54 (0.85–2.78)	0.16

*TIA* indicates transient ischemic attack, *PT* peripheral thromboembolism, *NVAF* non-valvular atrial fibrillation, *HR* hazard ratio, and *CI* confidence interval

## Discussion

In this report from CAFR, our data collected from 8529 NVAF patients demonstrated that in non--anticoagulated patients, risk of thromboembolic events was higher in PeAF than PaAF before adjusting confounders. However, this difference became not significant after adjusting age, sex, history of stroke, hypertension and vascular diseases. In contrast, in anticoagulated patients, thromboembolic risk did not differ between PaAF and PeAF before and after adjusting possible confounders.

This is one of the first comparisons of thromboembolic outcomes in different NVAF patterns in large Chinese population. As patients receiving catheter ablation treatment had a low incidence of stroke [25], we excluded those who received catheter ablation and with no AF recurrence, to avoid the dilution effect of low-risk patients. Our results strengthen the recommendation of current guidelines on stroke prevention for NVAF patients, suggesting choosing anticoagulation treatment should not base on the pattern of AF.

Current guidelines recommend that the pattern of AF should not be taken into account when assessing the stroke risk and deciding the choice for thromboembolism prophylaxis treatment in patients with AF [3, 4], despite that the burden of AF is higher in PeAF patients than that in PaAF patients. Whether AF pattern is associated with stroke risk has aroused wide concern over the recent years.

Clinical trial cohorts have reported contradictory findings. A sub-analysis of the Gruppo Italiano per lo Studio della Sopravvivenza nell’Infarto Miocardico-Atrial Fibrillation (GISSI-AF) trial [14] reported a similar rate of thromboembolic events in patients with PeAF and PaAF, with a much lower incidence among the overall population (0.97%) compared with our findings. In the Randomized Evaluation of Long-Term Anticoagulation Therapy (RE-LY) trial [17], the overall risk of stroke or systemic embolism in patients with paroxysmal, persistent, and permanent AF were similar, with rates of 1.32, 1.55, and 1.49% per year, respectively. In contrast, other trials have reported different results. In the Rivaroxaban Once-daily, Oral, Direct Factor Xa Inhibition Compared with Vitamin K Antagonism for Prevention of Stroke and Embolism Trial in Atrial Fibrillation (ROCKET-AF) study [13], patients with PeAF had higher adjusted rates of stroke or systemic embolism (2.18 vs. 1.73% per year, *P* = 0.048) and all-cause mortality (4.78 vs. 3.52, *P* = 0.006) compared with patients with PaAF. The same was found in SPORTIF (Stroke Prevention Using an Oral Thrombin Inhibitor in Atrial Fibrillation) III and V trials [11]. AF pattern was found to be an independent predictor of stroke in the sub-analysis of AVERROES (Apixaban Versus ASA to Prevent Stroke In AF Patients Who Have Failed or Are Unsuitable for Vitamin K Antagonist Treatment) and ACTIVE A (the Atrial Fibrillation Clopidogrel Trial With Irbesartan for Prevention of Vascular Events) trials [7], which only selected aspirin-treated NVAF patients.

Observational cohorts have also reported contradictory findings. The incidence of ischemic stroke adjusted by warfarin and other risk factors was similar in PaAF as in PeAF (2.6 vs. 2.9 per 100 patient years) from the Stockholm Cohort of Atrial Fibrillation [15]. In an Asian cohort, the crude event rate was 2-fold higher among the permanent NVAF patients (2.29%) than paroxysmal (1.16%) or persistent (1.20%) AF patients (*P* = 0.001), while after adjustment for warfarin use and risk factors, the hazard ratio for thromboembolism did not differ between paroxysmal and permanent groups [16]. In a Japanese study, Takabayashi et al. observed PaAF was independently associated with lower incidence of stroke/systemic embolism than sustained AF in patients with or without anticoagulants [8].

One of the possible reasons for the inconsistent results may be due to the use of antithrombotic therapy for preventing stroke, which will limit the outcome events and therefore, reduce the power to detect the difference of stroke incidence across AF patterns. Inconsistent anticoagulation strategy by design, imbalances of anticoagulant intensity and differences in the use of OAC rates may act as confounders. Different anti-platelet and/or anticoagulant therapy including aspirin, warfarin and new oral anticoagulants were used in various studies, with different efficacy in preventing thromboembolism. For example, the ACTIVE A and AVERROES trials [7] observed aspirin-treated NVAF patients and concluded different rates of ischemic stroke were 2.1, 3.0 and 4.2% per year for paroxysmal, persistent, and permanent AF respectively. In the ARISTOTLE (Apixaban for Reduction in Stroke and Other Thrombo-embolic Events in Atrial Fibrillation) trial [12], in which all of the patients were treated with warfarin or NOAC, the rate of stroke or systemic embolism was significantly higher in patients with persistent or permanent AF than paroxysmal group (1.52 vs. 0.98%, adjusted *P* = 0.015).

Of note, few studies observed thromboembolism incidence according to the stratified application of anticoagulants in different AF types. In our cohort, risk of thromboembolic events were compared between PaAF and PeAF patients based on OAC therapy. Univariate and multivariate analysis of Cox proportional hazards regression models were performed in the absence of OAC therapy to evaluate the predictive value of AF types more accurately. For non-anticoagulated patients, PeAF group demonstrated a trend towards worse outcomes, with higher incidences of stroke/TIA/PT, all-cause death and cardiac/non-cardiac death than PaAF patients. In OAC users, risk of outcomes was comparable between PaAF and PeAF groups. Age ≥ 75 yrs. and prior history of stroke/TIA/PT were independent predictors for thromboembolism, which was consistent with most of the prior studies. The Fushimi Atrial Fibrillation Registry (The Registry Study of Atrial Fibrillation Patients in Fushimi-ku) [8] reported a lower risk of stroke/systemic embolism in PaAF patients both in non-OAC/OAC users and confirmed PaAF was an independent predictor of lower stroke/systemic embolism risk. However, our data did not find the difference, although our sample size was larger, and patients in our study had higher proportion of PaAF patients and slightly lower CHADS_2_ /CHA_2_DS_2_-VAS_C_ score.

Other reasons may possibly explain the conflicting results in different studies. For example, different risk levels of the study population are relevant. The present study showed a majority of baseline variables were evidently different between PeAF and PaAF patients, with higher risk and more underlying comorbidities in PeAF type. PaAF group had a lower CHADS_2_ (PaAF vs. PeAF:1.7 vs.2.1, *P* = 0.000) and CHA_2_DS_2_-VASc score (PaAF vs. PeAF 2.9 vs. 3.3, *P* = 0.000), which was similar with ACTIVE-A and AVERROES [7], but lower than the results from ROCKET-AF [13] trial (mean CHADS_2_ score 3.5 for both types). Our data indicated significant variations of stoke risk factors between the two types. In the presence of known risk factors involved in CHA_2_DS_2_-VASc score, progression from sinus rhythm to PaAF or more sustained forms is frequently seen along with atrial electrical and structural remodeling. AF types reflect different states in the process of AF progression and may be the consequence of interaction between CHA_2_DS_2_-VAS_C_ components rather than the risk factor of stroke.

Different proportion of PaAF patients was included in previous studies. The proportion of PaAF (54.4%) among 8529 NVAF patients in our study was similar with that of the Loire Valley Atrial Fibrillation Project (58.4%) [18], but higher than most of other studies which recruited PaAF patients less than 50%, such as ACTIVE A and AVERROES trials (24%) [7], ROCKET-AF trial (17.6%) [13], ACTIVE W(Atrial Fibrillation Clopidogrel Trial With Irbesartan for Prevention of Vascular Events) Substudy (17.9%) [19], J-RHYTHM Registry (38.3%) [16] and Fushimi study (48,1%) [8]. In GISSI-AF trial [14], higher proportion of PaAF patients (62.5%), lower CHADS_2_ score (1.41 ± 0.84) and lower incidence of thromboembolic events (0.97%) were observed compared with our study. PaAF represented the early stage of AF progression, with lower AF burden than PeAF. The duration and frequency of AF episodes may have contributed to the conflicting results from prior reports.

Definitions of stroke were also different in previous trials, such as ischemic stroke (i.e. cardioembolic, atherothrombotic, or lacunar infarction) or hemorrhagic stroke, or both types. Different criteria of event ascertainment may lead to discrepancy in rate of stroke and systemic embolism. In the ARISTOTLE, Stockholm studies and Fushimi Registry [8, 12, 15], stroke endpoints were defined as a composite of ischemic and hemorrhagic stroke, while in our study and some others [14, 26], only ischemic stroke associated with AF was designed as clinical endpoints that may limit the number of events.

The event rates in our study were lower compared to that reported in other studies, despite the CHADS_2_ and CHA_2_DS_2_-VASc scores were similar [8]. The lower incidence of thromboembolic events may be attributed to our study being a cohort reflecting current practice, with blood pressure, cholesterol and other risk factors well controlled compared to prior studies. In patients with AF and hypertension, having any elevated BP measurements was independently associated with a higher risk of stroke or systemic embolism [27]. This is supported by the post-hoc analysis of GISSI-AF trial [14], where the event rate was only 0.97% per year in patients without anticoagulants, even lower than our study. We used an independent endpoint adjudication committee to validate stroke event, which is not the usual way in observational studies and excluded about one quarter of stroke events which is self-reported by patients while turned out not to be a true event. Multiple studies have shown a progressive decline in the incidence of thromboembolism in non-anticoagulated patients identified with NVAF over the past several decades, which is evident in the present study that reports crude incident rates of ~ 2/100 patient years for thromboembolism. The progressive decline in thromboembolism of non-anticoagulated NVAF patients is undoubtedly multifactorial and may be secondary to improved treatment of morbidities and also by early identification of lower risk NVAF patients.

We stratified OAC use in different groups and adjusted several risk factors of thromboemblism. Some residual confoundings might still remain even after multivariate adjustment, especially factors like obesity, sleep apnea and smoking etc. Previous studies consistently indicated AF burden detected by implanted devices was associated with an increased risk of ischemic stroke [28, 29], but it is not possible for us to further stratify the PaAF patients into different levels of AF burden to investigate the differences in risk. Future investigations are necessary to indentify the correlation between AF pattern, AF burden and thromboembolic events.

## Conclusions

Overall, in our large cohort of Chinese NVAF population, stroke, all-cause death and cardiac/non-cardiac death was higher in non-anticoagulated PeAF patients compared with PaAF group, but same between anticoagulated PeAF and PaAF patients. After adjustment, AF type was not an independent predictor of thromboembolism in NVAF patients.

## Data Availability

The datasets used and analysed during the current study are available from the corresponding author on reasonable request.

## Abbreviations

ACC: American college of cardiology
ACEI: Angiotensin converting enzyme inhibitor
AHA: American Heart Association
ARB: Angiotensin receptor inhibitor
BMI: Body mass index
CAFR: Chinese atrial fibrillation registry
CCr: Creatinine clearance
CHA_2_DS_2_-VAS_C_: Congestive heart failure, hypertension, Age ≥ 75 years, Diabetes, Stroke, Vascular diseases, Age65-74 years, sex category
CHADS_2_: Congestive heart failure, hypertension, Age ≥ 75 years, Diabetes, stroke
CI: Confidence interval
CNS: Central nervous system
CT: Computed tomography
DBp: Diastolic blood pressure
EHRA: European heart rhythm society
ESC: Europe society of cardiology
HR: Hazard ratio
HRS: Heart Rhythm Society
LA: Left atrium
LVEF: Left ventricular ejection fraction
NOAC: New Oral anticoagulant
NVAF: Non-Valvular atrial fibrillation
OAC: Oral anticoagulant
PT: Peripheral thromboembolism
SBp: Systolic blood pressure
SD: Standard deviation
TIA: Transient ischemic attack
